# Tuning Methylation-Dependent Silencing Dynamics by Synthetic Modulation of CpG Density

**DOI:** 10.1021/acssynbio.3c00078

**Published:** 2023-08-12

**Authors:** Yitong Ma, Mark W. Budde, Junqin Zhu, Michael B. Elowitz

**Affiliations:** † Division of Biology and Biological Engineering, California Institute of Technology, Pasadena, California 91125, United States; ‡ Primordium Labs, Arcadia, California 91006, United States; § Department of Biology, Stanford University, Stanford, California 94305, United States; ∥ Howard Hughes Medical Institute, California Institute of Technology, Pasadena, California 91125, United States

**Keywords:** epigenetics, DNA methylation, DNMT3b, synthetic biology

## Abstract

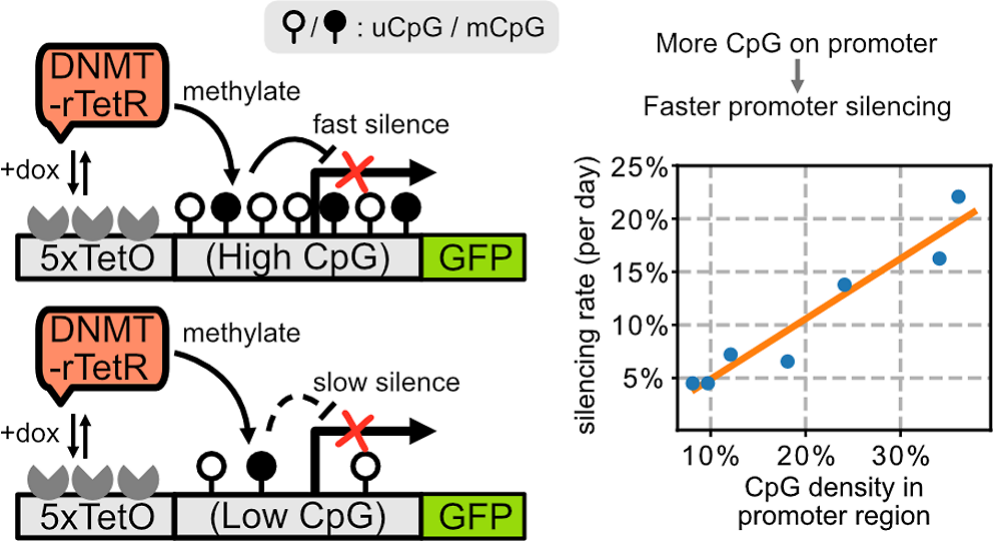

Methylation of cytosines in CG dinucleotides (CpGs) within promoters has been shown to lead to gene silencing in mammals in natural contexts. Recently, engineered recruitment of methyltransferases (DNMTs) at specific loci was shown to be sufficient to silence synthetic and endogenous gene expression through this mechanism. A critical parameter for DNA methylation-based silencing is the distribution of CpGs within the target promoter. However, how the number or density of CpGs in the target promoter affects the dynamics of silencing by DNMT recruitment has remained unclear. Here, we constructed a library of promoters with systematically varying CpG content, and analyzed the rate of silencing in response to recruitment of DNMT. We observed a tight correlation between silencing rate and CpG content. Further, methylation-specific analysis revealed a constant accumulation rate of methylation at the promoter after DNMT recruitment. We identified a single CpG site between TATA box and transcription start site (TSS) that accounted for a substantial part of the difference in silencing rates between promoters with differing CpG content, indicating that certain residues play disproportionate roles in controlling silencing. Together, these results provide a library of promoters for synthetic epigenetic and gene regulation applications, as well as insights into the regulatory link between CpG content and silencing rate.

## Introduction

Methylation of CG dinucleotides (CpGs) plays critical roles in mammalian development, tumor progression, and aging.^1−4^ These functions result mainly from the ability of CpG methylation to induce and stabilize gene silencing in mammals through multiple mechanisms.^5,6^ Control of DNA methylation and further gene silencing depends on both trans-acting factors and the DNA sequence itself. Trans-factors include methylation “writers” such as DNA methyl-transferases (DNMTs)^7^ and “erasers” such as TET1^8^ that establish and alter methylation marks, as well as “readers” such as MeCP2 and histone deacetylases^9^ that link methylation to regulation of gene transcription.^6,10^ In mammalian cells, DNA methylation occurs mainly at CpGs. As a result, the distribution of CpG dinucleotides within a given sequence can play a pivotal role in methylation-based gene regulation.

At the genome level, regions with different CpG content exhibit distinct methylation patterns,^11,12^ potentially due to cooperativity between nearby CpGs and other suppressive epigenetic marks, which can generate positive feedback.^13,14^ Relatively high CpG-density regions (CpG islands) from a human chromosome largely maintain their methylation state when hosted in a transchromosomic mouse model,^15^ suggesting that DNA sequence composition plays a strong role in establishing stable methylation states. Conversely, insertion of several hundred base pairs of CpG-free DNA can disrupt these patterns, permitting *de novo* methylation of the surrounding CpG island.^16^ However, the precise role of CpG sequence context can be difficult to discern at natural loci, where regulation is also affected by many other cis- and trans-acting factors, including cell-type specific methylation writer and reader profiles, neighboring (non-CpG) motifs that recruit epigenetic modifiers, pre-existing chromatin states, and so forth. To clearly delineate the role of DNA sequence in methylation and silencing, one would ideally want to directly compare the methylation and silencing of similar promoters with different CpG distributions, in the same genomic context. Further, because methylation and its effects on gene regulation can both be dynamic,^17−19^ the ability to control the timing of methyltransferases (DNMTs) recruitment to a locus and follow the resulting changes in gene expression is also desirable.

The field of synthetic epigenetics seeks to harness epigenetic regulatory mechanisms to control gene expression on different timescales.^20,21^ Recent work demonstrated the ability to regulate synthetic or endogenous gene expression by recruiting DNMTs to specific target genes^18,22−24^ and even create fully synthetic DNA methylation-based systems for synthetic epigenetic memory.^25^ In CHO-K1 cells, transient DNMT recruitment to a locus drives stochastic, irreversible, all-or-none silencing over timescales of about 5 days.^18^ However, it is unclear how the dynamics of gene silencing depends on the DNA sequence of the regulated target gene. Understanding the effects of sequence composition on silencing rates could provide insights into gene regulation by DNA methylation and also expand the synthetic epigenetic toolbox for fine tuning circuits.

Here, we adapted a previously established DNMT recruitment system to analyze the effects of DNA sequence on methylation-dependent silencing.^18^ We derived a library of promoters with different CpG densities from a synthetic promoter and observed the relationship between CpG density and the silencing dynamics occurring after DNMT recruitment. Using a mathematical model of methylation, together with sequencing identifying methylation marks, we showed that the observed gene expression dynamics could be explained by methylation accumulation on the DNA. We also identified several specific CpG elements that appear to play disproportionate roles in silencing dynamics and confirmed that one of them [near the TATA box and transcription start site (TSS)] causes significant changes in silencing dynamics. Our results reveal how CpG density influences silencing dynamics and provide a library of promoters with different silencing rates for synthetic applications.

## Results and Discussion

### Construction of a Promoter Library with Varying CpG Content

To investigate the relation between promoter CpG content and its DNMT-dependent silencing rate, we adopted a previously described synthetic methylation-silencing system.^18^ In this system, the catalytic domain of DNMT3b (DNMT3bCD) is fused with a reversed tetracycline repressor (rTetR), allowing precise temporal control of the recruitment to a target gene by adding doxycycline (dox) to the culture media.^26^ Here, to focus on the role of DNA methylation in silencing, we specifically used the mouse gene Dnmt3b′s catalytic domain, spanning amino acids 402–872. This region omits the major heterochromatin-interacting PWWP domain.^27^ This construct also incorporates a co-expressed H2B-mCherry fluorescent protein fusion. We stably integrated this construct using the piggyBac transposon system and sorted cell populations with similar mCherry fluorescence level (Figure S1A). This enabled direct comparison of different promoters (see below) with the same DNMT3bCD expression context.

**Figure 1:**
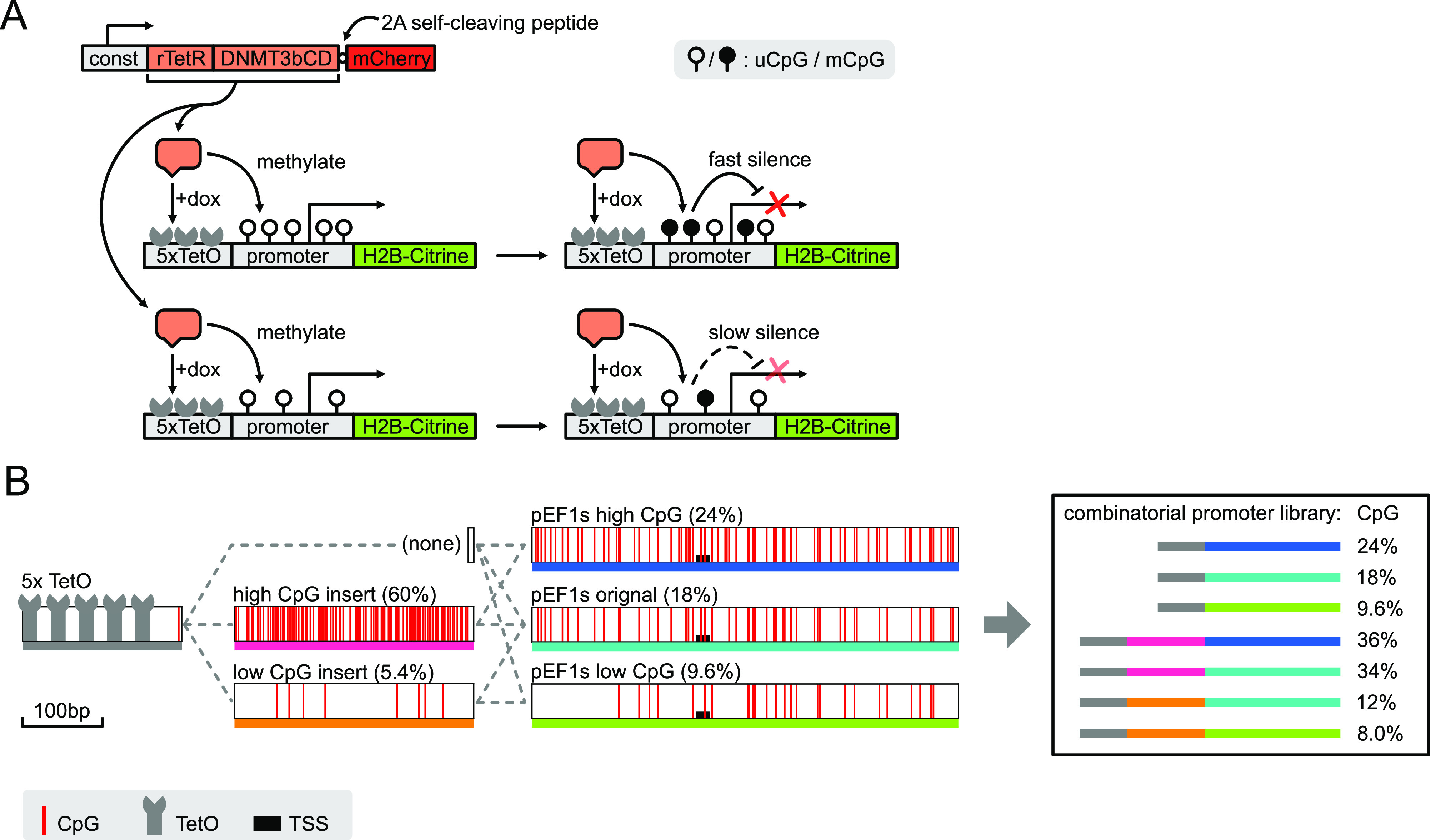
rTetR fused DNMT3b catalytic domain methylates and silences a reporter library of different CpG content upon recruitment: (A) schematic of the synthetic methylation-silencing system. rTetR fused DNMT3bCD is expressed constitutively, and upon induction of dox, recruited to the promoter region of a site-specifically integrated Citrine reporter. The recruitment methylates the promoter and further silences the gene expression, with dynamics depending on the promoter’s CpG content. (B) Design of the library of promoters with varying CpG content. 5× tandem TetO binding sites were fused with an insert (or no insert) and a pEF1s synthetic promoter to make the library of promoters. Red lines represent CpG dinucleotides, and the pEF1s promoters are vertically aligned to show sequence homology.

We used an H2B-mCitrine^28^ fluorescent fusion protein as the target gene. This target was driven by one of a set of promoters containing varying densities of CpG (see below). In each promoter, an array of 5 rTetR binding sites (TetO) was fused upstream of the promoter, allowing recruitment of rTetR-DNMT3bCD (Figure 1A). To enable direct comparison between target promoters at the same genomic context, all reporter cassettes were site-specifically integrated as a single copy into the epigenetically active φC31attP/attB landing site within an artificial chromosome that was previously engineered into CHO cells (MI-HAC CHO cells, also see Materials and Methods).^29^

We constructed a library of synthetic promoters that differed in their CpG densities. We started with a synthetic version of the human elongation factor 1α promoter [pEF1s(orig), with 18% CpG density], a 544 bp fusion of promoter fragments from the human EF1α promoter and human T-cell virus (HTLV),^30^ that is commercially available (InvivoGen) and has been used for antibody expression and gene therapies.^31,32^ To identify conserved CpG elements, we compared both the EF1α fragment and HTLV fragments of this promoter to their natural orthologs, respectively.^33^ We then removed or added CG pairs into the promoter at non-conserved sites. With this procedure, we generated promoters with varying CpG densities at 9.6 and 24% [pEF1s(low) and pEF1s(high), respectively, Figure 1B, middle].

Next, to broaden the range of CpG densities, we designed an additional DNA segment, inserted upstream of the promoter, containing high (60%) CpG density (Figure 1B, high CpG insert). We altered this CpG insert by swapping out CG with GC dinucleotides, or by replacing C with T, to create a lower CpG density (5.4%) insert, while otherwise preserving its sequence similarity with the high CpG insert (Figure 1B, low CpG insert). Altogether, we combined the three pEF1s promoters with the two inserts, or with no insert, to produce a library of 7 sequences whose overall CpG density ranged from 8.0 to 36% (Figure 1B right). Despite their variation in CpG density, all 7 promoters drove strong expression of the fluorescent protein reporter, producing ∼200-fold greater signal compared to autofluorescence in non-transfected cells, with a 2.8 fold variation of expression level (Figure S1B,C). This difference could be either due to the difference in the promoter sequences or due to altered mRNA secondary structure of 5′UTR (part of the altered promoter sequence), resulting in changes in RNA half-life.^34^ However, this expression change did not impact quantification of the silenced fraction, as silencing thresholds were set independently for each promoter (see below). As expected, the original pEF1s promoter shows the highest activity, while our alterations to the sequence (both addition or reduction of CpGs) slightly lowered its activity. Therefore, there was no correlation between expression level and CpG content.

### DNMT-Dependent Silencing Rate Correlates with Promoter CpG Density

**Figure 2:**
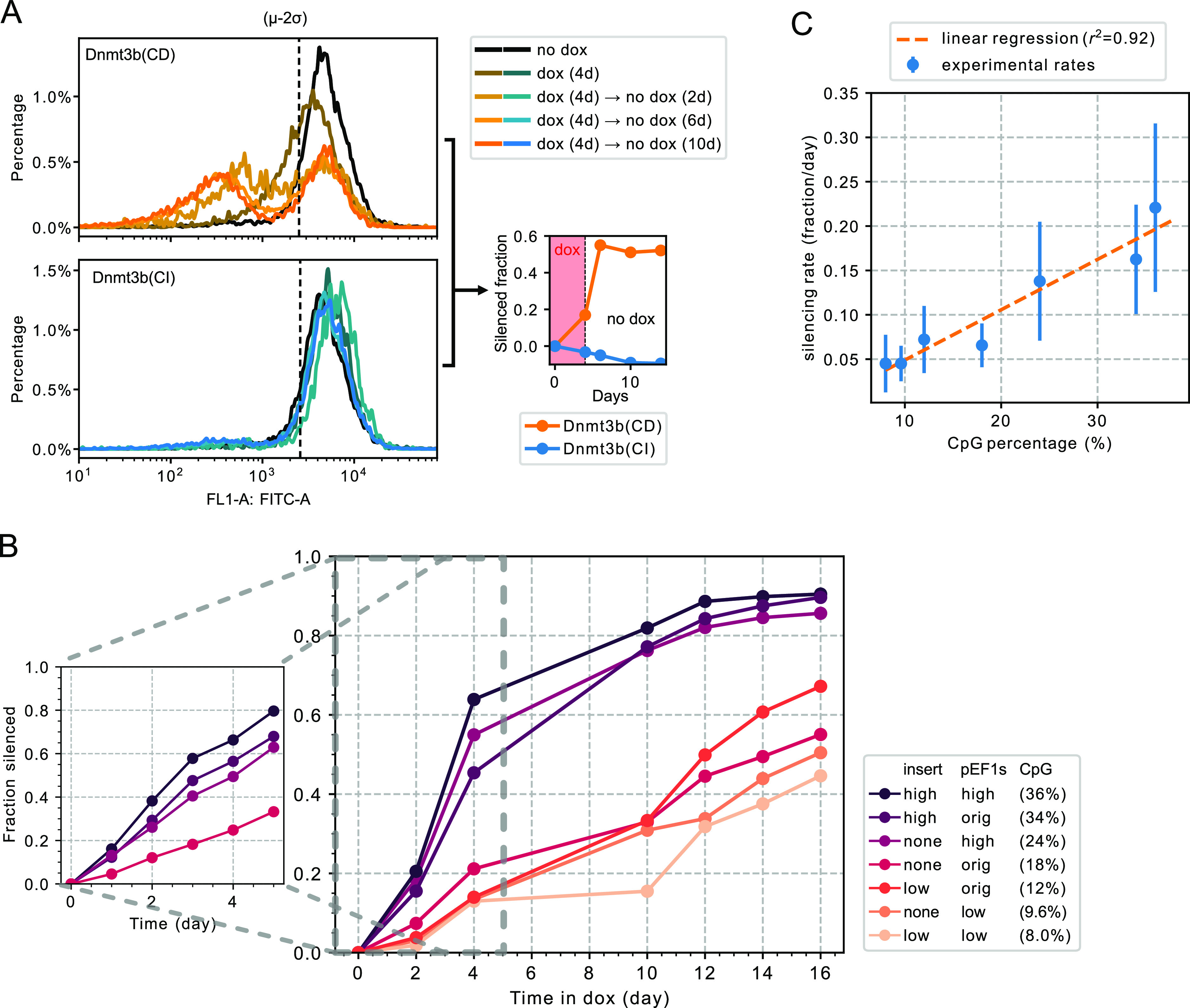
Promoters’ silencing rate correlates with their CpG content. (A) Promoter silences with all-or-none kinetics when DNMT3bCD is recruited to the locus, and this silencing is dependent on DNMT3b’s catalytic activity. Cells with the pEF1s(high) promoter were treated with dox for 4 days, and then no dox (release) for 2, 6, and 10 days. Cells are analyzed by flow cytometry at various time points (left) and quantified by comparing to the no dox control (black lines in the left). A log–normal distribution is first fitted onto the no dox control’s positive population, and μ–2σ are used for quantification of silenced fractions. These fractions are then normalized to no dox controls’ silenced fraction (see Materials and Methods, right). The silenced fractions are stable after the release of dox for 2 days, and no silencing is observed in the DNMT3bCI controls. (B) Time course of the silenced fraction of different promoters. Cells were treated with or without dox, and then with 2 days of no dox (release). The fraction of silencing is determined as described in (A): cells with lower fluorescence than μ–2σ of the no dox control group were determined as silenced. The silencing rate is further normalized to the no dox group (see Materials and Methods). For the shorter time scale (left), the same method is used except with a higher time resolution. (C) Summary of the silencing rates in (B). The silencing rate is calculated by subtraction between each pair of neighboring dots and then normalized by time intervals in between, as well as the remaining fraction size. We omitted dot pairs over 80% fraction as the normalization fraction is too small.

Previous analysis of silencing dynamics by DNA methylation in a similar system revealed that transcriptional silencing occurs through stochastic, all-or-none, irreversible events in individual cells.^18^ To confirm that our system has similar kinetics, we induced DNMT3bCD recruitment to promoters for 4 days and then released the recruitment for 2, 6 and 10 days and measured the Citrine fluorescence via flow cytometry for all three promoter variants [Figures 2A and S2A,B for pEF1s(high), pEF1s(orig), and pEF1s(low), respectively]. As expected, a fraction of the cells were transcriptionally silenced after the induction, and gradually diluted out stable H2B-Citrine fluorescent protein during the “release” phase due to cell divisions (approximately 22 h per division, observed by the shifting position of silenced population peaks). Meanwhile, the active populations (peaks on the right) remained stable in terms of both fluorescence level and cell population fraction, indicating an all-or-none, irreversible kinetics as reported before.

Setting a silencing threshold 2 standard deviations (2σ) below the mean fluorescence levels of actively expressing (“no dox”) cells yielded a stable value after 2 days of release (see quantification on the right in Figures 2A and S2A,B), allowing consistent quantitation of all-or-none silencing. Because the control groups were single peaked and exhibited consistent variation, this cutoff occurred at values ranging from 54 to 71% of the mean, depending on cell line (Figure S2C).

We also used a catalytically inactive version of the DNMT3bCD protein (P656V and C657D double point mutations,^35^ noted as DNMT3bCI) as a negative control. Even though the CI versions were expressed at a similar level of the CD version (Figure S1A lower half), no silencing effect was observed when they were recruited to the promoters (Figures 2A and S2A,B, lower half as well as blue lines in the quantifications). This confirmed that the observed silencing effects resulted specifically from DNA methylation activity and not from other protein interactions or interference with transcription machinery.

These results established that the promoters silence in a methylation-dependent, all-or-none and irreversible manner and indicate that silencing kinetics can be captured by the dox induce-and-release protocol.

To quantify silencing dynamics across the library, we analyzed the dynamics of silent cell accumulation over a time course (up to 19 days) of dox induction with 2-day subsequent release of the recruitment at various time points (Figure 2B, right). As some of the promoters, notably those with insert(high) or pEF1s(high), reached over 50% of silencing only after four days (about two time points in our setup), we added a biological replicate for each of the four fastest promoters with a separate time course with finer time resolution (Figure 2B, left). From the time course, we can conclude that higher overall CpG density on the promoter results in faster silencing dynamics. This result is robust to analysis with a stricter cutoff of 90% expression reduction (Figure S2B). Even with the less sensitive threshold, we observed a similar trend of increased CpG density correlating with faster silencing.

The time-course dynamics for each promoter could be summarized by an empirical silencing rate as the silenced fraction per unit time (day), normalized by the remaining active population. Silencing rates varied over nearly an order of magnitude across promoters with various CpG densities. Further, the silencing rate correlated linearly with CpG density over this range (Figure 2C). These results showed that across varying CpG densities, DNMT-dependent silencing dynamics are broadly consistent with a stochastic, all-or-none silencing process, occurring at a rate that depends on CpG density.

### Silencing Kinetics Follow a Stochastic Switching Model

Previous studies using a similar system with a different version of the EF1α promoter showed that silencing kinetics could be described as a single-step stochastic switching event from the active to the silent state^18^ (Figure S3A). In the model, each promoter silences stochastically at a time-invariant rate β. Here, we tested whether a similar model could fit our data, if we allowed β(*c*) to depend on CpG density, *c*. With this assumption, the size of the active population fraction, *A*, can be described by a simple differential equation
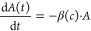
1

Note that cell proliferation does not need to be explicitly incorporated due to the heritability of the expression state. In this model, the active population, *A*, decays exponentially over time, *t*. To test this model, we plotted the time course data (Figure 2B) in terms of the remaining active population, *A*(*t*) (Figure S3B), and observed a linear–log relationship, consistent with exponential decay, for every promoter variant. In these plots, the switching rate β(*c*) ranged from 0.032 to 0.274 d^–1^, depending on CpG density. These results are consistent with a simple stochastic switching model in which silencing rate is tuned by CpG density.

### Methylation Accumulates after DNMT Recruitment

**Figure 3:**
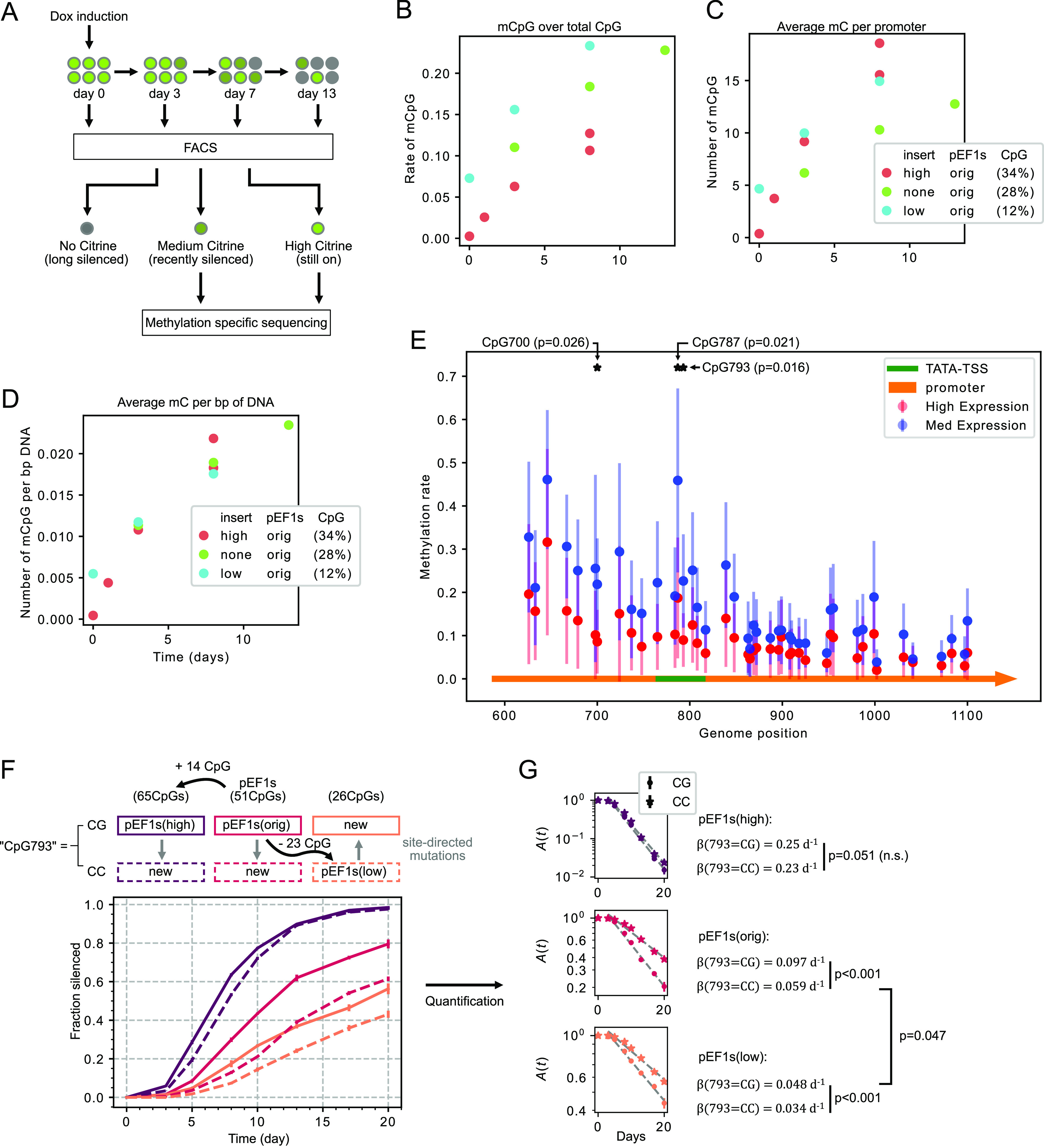
Sequencing reveals the constant accumulation of methylation and, potentially, master CpGs. (A) Schematics of the FACS-Sequencing experiment: cells with different promoters are treated with dox, and then FACS-sorted to three bins based on the Citrine brightness (high, med, and low), consisting cells that are “still ON,” “recently silenced,” and “long silenced,” respectively. The first two groups proceed to downstream methylation-specific sequencing (see Materials and Methods). (B–D) Total CpG methylation (B), methylation frequency (C), and total methylation normalized by promoter length (D) accumulates in the promoter with time in the “still ON” cell population. “Still ON” populations are sorted out as indicated in (A) at intended dates, and subsequently analyzed by methylation-specific sequencing (EM-seq), targeting the integrated gene promoter. (E) CpGs around TATA-box and TSS (highlighted in green) show significant difference in methylation between the “still ON” and “recently silenced” group. Methylation percentages of different samples at different days were pooled together for comparison (a total of 10 from “still ON” group compared to 6 from “recently silenced” group). *P*-values are from Student *t*-test. (F) Mutation at CpG793 changes promoters’ silencing rate significantly. New cell lines are constructed by introducing point mutations (CG to CC or the inverse) at CpG793 in pEF1s(high), pEF1s(orig), and pEF1s(low) promoters (top). Cell lines are then constructed as described previously in this paper. DNMT3bCD recruitments are induced by dox at day zero and cells were analyzed by flow cytometry at each time point after dox induction. (G) Quantification of the silencing rate (similar method as Figure S3A and eq 1) of time course in (F). We excluded the time point at 0 from the fitting as dox release was not included in this experiment. *P*-values are calculated based on the estimation and standard error of β from linear regression.

Given that promoter silencing depends on the methylation activity of the recruited DNMT3bCD, we next asked whether methylation accumulates at similar or different rates for different promoters. We used fluorescence activated cell sorting (FACS) to isolate the transcriptionally active cell fraction (*A* in the model) at different times after dox addition (Figure 3A). We then measured promoter CpG methylation profiles using methylation-specific sequencing (EM-seq^36^) (Materials and Methods, Figure 3A).

As expected, methylation accumulated in the transcriptionally active populations, as measured by methylation rate (methyl-CpG over total CpG), total methylation per promoter, as well as total methylation per promoter per bp of DNA (Figure 3B–D respectively). Unexpectedly, however, the rate of methylation accumulation was independent of CpG density, measured as total methylation per bp of DNA (Figure 3D). In fact, the rate of methylation per CpG was greater at promoters with lower CpG densities (Figure 3B), while the total number of methylated CpG in the promoter region was similar across different promoters. This behavior is compatible with saturation of methylation capacity of the locally recruited DNMT3bCD. Alternatively, it could also reflect an effective interaction, in which unmethylated CpGs inhibit methylation at nearby CpG sites.^13^

### Single CpG Has a Disproportionate Impact on Silencing Rate

The apparent discrepancy between the CpG density-dependent silencing rate and the density-independent methylation rate provoked the question of whether certain individual CpGs might play disproportionate roles in controlling silencing. Such CpGs would be expected to exhibit significant differences in methylation between cell populations containing active versus recently silenced promoters.

To discover such CpGs, we pooled all available sequencing results from different time points. Within the pEF1s region, where all three promoters overlap (∼90% of the pEF1s region) (Figure 3E), we identified three CpGs with significantly different methylation levels between the two expression groups (*p* < 0.05). Interestingly, two of the most significant CpGs, including the top ranked one (CpG at position 793, or CpG793 for short), are located between the TATA box and the TSS (arrow in Figure 3C), consistent with previous reports suggesting functionally important CpG islands around the TSS.^37^ CpG793 was among the CpGs that were eliminated in the construction of the low CpG pEF1s(low) promoter, consistent with the lower silencing rate observed for this promoter (Figure 2B).

To test for a functional role of CpG793, we mutated it to CC in pEF1s(orig) and pEF1s(high). Conversely, we also reverted this position back to CG in pEF1s(low), where all 22 other CpGs including CpG793 were mutated previously. Together, these constructs provided a set of controlled comparisons in which position 793 was either CC or CG in pEF1s(high), pEF1s(orig), and pEF1s(low) (Figure 3F, top).

We analyzed silencing rates (fraction per day normalized by remaining fraction, similar to Figure S3B) for each of these promoters. These rates were significantly reduced in the “CC” variants of pEF1s(orig) (*p* < 0.001) and pEF1s(low) (*p* < 0.001), but not the pEF1s(high) promoters, compared to the CG variants (Figures 3F lower, and 3G). Further, silencing rates were similar between the CG variant of pEFs1(low) and the CC variant of pEFs1(orig) (barely significantly different with *p* = 0.047), even though these two sequences systematically differed at 22 other CpGs. This indicates that the position 793 mutation could almost compensate for the combined effect of 22 other CpG mutations.

Finally, we asked if the observed differential silencing dynamics caused by the CC-CG mutation at position 793 could result from disruption or introduction of a known transcription factor binding site. We queried the surrounding sequence (±8 nt, 18 nts in total) against known mouse cis-regulatory elements in CIS-BP^38^ and filtered for hits expressed in CHO cells^39^ based on criteria suggested previously^40^ (Table S1). The only hits observed in both the “low” and “orig” promoters were Gmeb1 and Gmeb2, a pair of proteins that are involved in modulating glucocorticoid receptor-mediated transactivation.^41^ However, these proteins are not known to be directly involved in epigenetic regulation, to our knowledge. While we cannot rule out the possibility that the observed difference in silencing results from differential binding of sequence-dependent cis-factors, it is consistent with the explanation that methylation capability at this position has a disproportionate effect on silencing.

Together, we observed three key results: First, CpG methylation in promoters in the “still ON” population accumulated with time. Second, the silencing rate did not correlate with either methylation rate or total methylation, contrary to expectation. Third, we discovered a specific CpG position that plays a disproportionate, functional role in controlling silencing rate.

## Conclusions

While effects of sequence on DNMT-dependent gene silencing have long been observed, a controlled system for directly analyzing the effects of sequence on silencing has not been available. Here, we constructed a library of synthetic promoters, featuring varying CpG content and methylation-dependent silencing kinetics (Figure 1). Strikingly, silencing rate correlates directly with CpG content (Figure 2C). However, this correlation could not be explained by a corresponding effect of CpG content on methylation, as methylation accumulated at similar rates in all promoter variants (Figure 3B–D). Finally, we observed evidence that a certain CpG (CpG793), located between the TATA box and the TSS, can play a disproportionate role in control of silencing rate (Figure 3F,G). Together, these results should provide a versatile set of components for engineering synthetic epigenetic circuits with desired silencing behaviors, as well as a foundation for future investigations of the mechanisms of DNMT-dependent silencing. Finally, our observation that the DNA sequence-based substrate of epigenetic modifications could alter the regulation dynamics might also apply into fully synthetic epigenetic circuits.^25^

A remaining mystery is why the rate of methylation accumulation is correlated neither with the rate of silencing nor with the CpG content of the promoter. Despite the lack of correlation between silencing rate and accumulated methylation, promoter silencing depended on the methylation activity of the recruited DNMT, as a catalytically inactive variant of DNMT3b was not able to initiate silencing in our system (Figure 2A), indicating that *de novo* DNA methylation is a necessary requirement for promoter silencing in this context.

A possible explanation could be that silencing requires at least two distinct steps, mediated by two types of trans-regulatory factors: the first binds to methyl-CpG, and the second binds to CpG in a methylation-independent fashion. If only a small number of methyl-CpG is required for the first, methyl-dependent factor(s), then total CpG density could establish a rate-limiting step for advancing to a silent state. Examples of both types of proteins exist. Methyl-binding domain (MBD) proteins like MeCP2, MBD2, and so forth are known to play key roles in methylation-dependent silencing.^6,42^ At the same time, CpG islands are known to be able to initiate silencing by recruiting polycomb group proteins independent of methylation in embryonic stem cells differentiating into neurons.^43^ There are also “dual functional” proteins (e.g., TET1^44^ and KDM2B^45^) that bind to un-methylated CpGs but still promote gene silencing in some cell contexts. Further experiments could help to disentangle the roles of methylation-dependent and independent factors in controlling the rate of silencing.

Starting within two days after the release of dox, the active population remained in an actively expressing state (Figure 2A). DNA methylation is actively maintained and, thus, unlikely to dilute out during this period without active recruitment of de-methylation enzymes.^46^ Therefore, the recruited DNMT3bCD protein may play an additional role in silencing beyond its catalytic activity as a methyltransferase. In fact, full length DNMT3b, even with its catalytic domain deactivated, has significant functions in epigenetic gene regulation through its protein and heterochromatin interacting domain.^35^ In this study, we specifically recruited the DNMT3b “catalytic domain” (with the PWWP domain deleted). However, this protein still includes the ATRX domain that has been shown to associate with heterochromatin.^27^ Further investigation will be needed to identify the roles of methyltransferase-dependent and independent activities of DNMT3b.

One factor that could complicate our comparison between promoters is the distance from the recruited site (5xtetO) to the promoter’s core. This distance differed in promoters with additional inserts. However, the correlation of silencing rate with CpG density occurred among groups of constructs either lacking or containing the insert, when these groups were considered separately. This suggests that the change in distance to the core promoter (roughly 300 base pairs) in this system is not responsible for silencing rate correlation.

Additionally, our discovery of CpG793 playing a disproportionate role in determining silencing dynamics also suggested our model of correlation between CpG density and silencing rate is incomplete. A much larger set of promoter variants containing combinatory mutations on all CpGs may provide a more complete model accounting for the individual effects of each CpG. We believe this issue would be better resolved in the future using a massively parallel reporter assay approach that can access much larger numbers of promoters.

Finally, we note that phenotypically, our findings resemble the genetic mechanism of fragile X syndrome (FRX), in which an increased CGG repeat number upstream of the FRM1 gene’s promoter leads to hypermethylation and gene silencing during development.^47^ The exact molecular mechanism leading to silencing in FRX is not yet fully understood, but various hypotheses, including toxic secondary RNA structure^48^ and aberrant histone deacetylation,^49^ have been proposed. It would be interesting to find out to what extent the mechanisms underlying the relationships observed here may be shared with those involved in FRX.

## Materials and Methods

### Cell Culture Maintaining

CHO cells containing a human artificial chromosome (CHO-HAC)^29^ were cultured at 37 °C, in a humidified atmosphere with 5% CO_2_. The growth media consisted of Alpha MEM Earle’s Salts (Irvine Scientific) with 10% Tet Approved FBS (Clontech Laboratories or Avantor) and 1× penicillin/streptomycin (Life Technologies) and 1× GlutaMax (Gibco) added. Cells were passaged according to the standard CHO-K1 cell (CCL-61, ATCC) procedure.

### Plasmid and Cell Line Construction

All plasmids are constructed using standard cloning techniques, including Gibson Assembly (NEB) and GoldenGate Assembly (NEB). The plasmids and their maps are available for requests at Addgene (addgene.org/browse/article/28233817/).

The basal cell line expressing rTetR-DNMT3B (CD and CI version) was constructed by transfection and stable integration via the PiggyBAC system (System Biosciences), following manufacturer’s instructions, followed by blasticidin (Gibco) selection at 10 μg/mL for 5 days. The cells were then sorted for similar mCherry expression (Figure S1A), or single cloned further reporter integration (in the case of finer time course in Figure 2B). For integration of the reporter, methods similar to previous literature^18^ were used. Briefly, we co-transfected 600 ng reporter plasmid and 200 ng PhiC31 integrase plasmid using Lipofectamine 2000 (Invitrogen). After selection by geneticin (Gibco) at 400 ng/mL for 14 days (Figure S1B), cells were sorted (see below) to isolate the population with expression around the highest peak (Figure S1C, expected expression of single integration, as their system’s single integration rate should be close to 90% after selection^29^).

### Flow Cytometry and FACS

Cells were washed by PBS, lifted by 0.25% EDTA–Trypsin (Gibco), and diluted in HBSS (Gibco) with 0.25% of BSA before flow cytometry. Flow cytometry experiments were performed either on MACSQuant VYB Analyzer (Miltenyi Biotec) or CytoFLEX (Beckman Coulter). Analysis of data was done with open source, in-house developed software, EasyFlow (https://github.com/AntebiLab/easyflow), or EasyFlowQ (https://github.com/ym3141/EasyFlowQ).

FACS was performed with SY3200 Cell Sorter (Sony) at Caltech FLow Cytometry Facility.

### Enzymatic Methylation-Specific Sequencing and Analysis

Cells were sorted as described above and immediately lysed for DNA extraction (DNeasy Blood & Tissue Kit, Qiagen). Total DNA was then converted with NEBNext Enzymatic Methyl-seq Conversion Module (NEB) according to the manufacturer’s instructions and further amplified (EpiMark Hot Start Taq DNA Polymerase, NEB) with primers targeting a 2.5 kb region containing TetO binding sites, promoter region, and the gene body (nucleotide 1943–4438 on the none-pEF1s(orig) plasmid). The amplified targets were further prepared into library (Nextera XT Library Prep protocol Illumina) and sequenced on the MiSeq (250 bp pair ended, Illumina) platform.

The resulting reads were first trimmed and filtered by Trim Galore! (Babraham Institute) and then aligned and analyzed by Bismark^50^ and SAMtools^51^ to generate the methylation calling statistics.

### Data Processing and Statistical Testing

For calculating the silenced fractions, the background silenced fraction (*S*_dox–_) from the no recruitment control sample (no dox) was subtracted from the observed silenced fraction (*S*_dox+_) from the with recruitment group experiment and further normalized by the “fraction still available for silencing” (“still ON” fraction in the control 1 – *S*_dox–_. Consequently, the silenced rate of a given sample was calculated as follows:
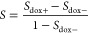


All statistical testings in this study were Student’s *t*-test if not specified.

Error bars in Figure 3F,G were generated via bootstrapping. Specifically, for each time point in Figure 3F,G, each of the three “with recruitment” samples were normalized to each of three “no recruitment” control samples, according to the method described above. Therefore, a total of 9 data points were generated, and the error bars represent the standard deviation of these points.

## Supporting Information Available

Legend for transcription factors that bind differentially only to the CG or CC (at CpG793) version of the promoters; additional characterization of the cell lines; additional characterization of the silencing time course and analysis with alternative criteria; and mathematical model schematics and fittings to the data (PDF)

## Notes

Plasmids and their maps available for requests at Addgene (addgene.org/browse/article/28233817/). The key cell lines are available upon request. EM-Seq raw and processed data is deposited at Gene Expression Omnibus (GSE224403). Data and codes for analysis and generating figures are available at data.caltech (doi: 10.22002/ct5kt-cv878).
