# Supplementary material for: Tuning Methylation-Dependent Silencing Dynamics by Synthetic Modulation of CpG Density
Source: ACS Synth Biol. 2023 Aug 12;12(9):2536–45. doi: 10.1021/acssynbio.3c00078 (PMC10510725; doi:10.1021/acssynbio.3c00078)
Supplement: Supplementary file 1 — sb3c00078_si_001.pdf [file sb3c00078_si_001.pdf]

Supporting information

Tuning methylation-dependent silencing dynamics by synthetic modulation of CpG density

Yitong Ma[1], Mark W. Budde[1,2], Junqin Zhu[3], Michael B. Elowitz[1,4,*]

1. Division of Biology and Biological Engineering, California Institute of Technology, Pasadena, CA 91125, USA
2. Primordium Labs, Arcadia, CA 91006, USA
3. Department of Biology, Stanford University, Stanford, CA 94305, USA
4. Howard Hughes Medical Institute, California Institute of Technology, Pasadena, CA 91125, USA

*Correspondence: melowitz@caltech.edu

Supplementary table

	pEF1s(low)	pEF1s(orig)	pEF1s(high)
CG variant	Tfe3, Tfeb, Mitf, Arntl, Mlx, Gmeb1, Gmeb2, Usf2*	Gmeb1, Gmeb2	Dnmt1*, Kdm2b, Kmt2a
CC variant	Zbtb7bm	-	-

Supplementary Table S1: Transcription factors that bind differentially only to the CG or CC (at CpG793) version of the promoters.
Surrounding sequences (±8 nt, 18 nts in total) of CpG793 in three different promoters were queried against known mouse transcription factors (CIS-BP[1]) for potential differential TF binding between the CG and CC version. Potential hits were then compared to wild type CHO's transcriptome data[2], Genes that cannot be mapped in CHO transcriptome, or have a FKPM smaller than 1 were filtered out[3]. Among the hits, high expression genes (greater than 1/100 of the housekeeping Actb gene) are annotated with stars.

Supplementary figure and legends

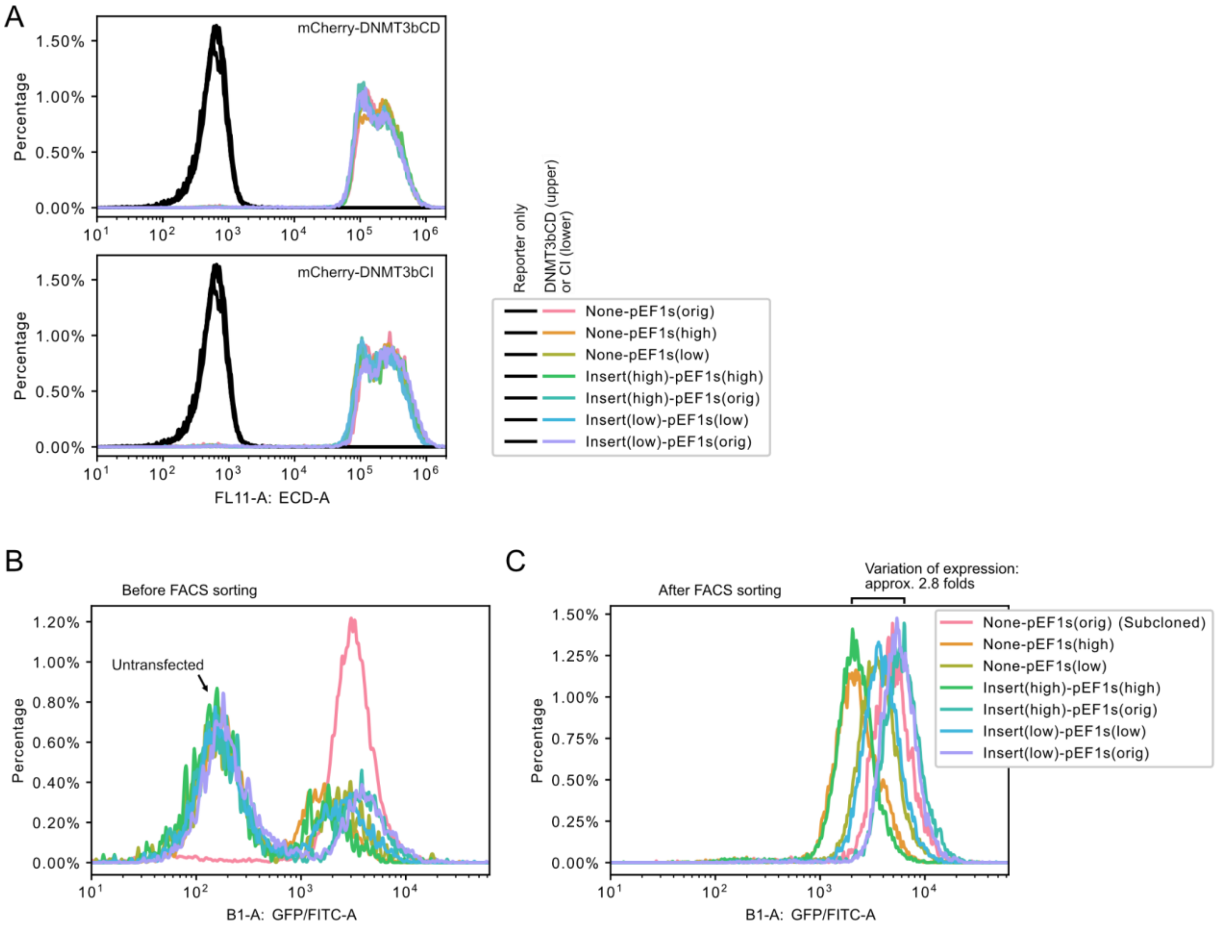

Figure S1. Generation of the cell line.

- *A.* *Cell lines with different promoters had similar expression levels of DNMT3bCD (top) or DNMT3bCI (bottom). Cell lines that were transfected with DNMT3b (CD or CI) with co-expressing mCherry fluorescent protein, were selected by blasticidin and then sorted (see MATERIALS AND METHODS).*
- *B-C.* *Site-specific integration of the reporters generates cell populations with uniform expression after FACS sorting. Cells are transfected and selected with geneticin for 14 days (B) (see MATERIALS AND METHODS), and then subcloned [None-pEF1s(orig)] or FACS sorted (all others) for further analysis (C).*

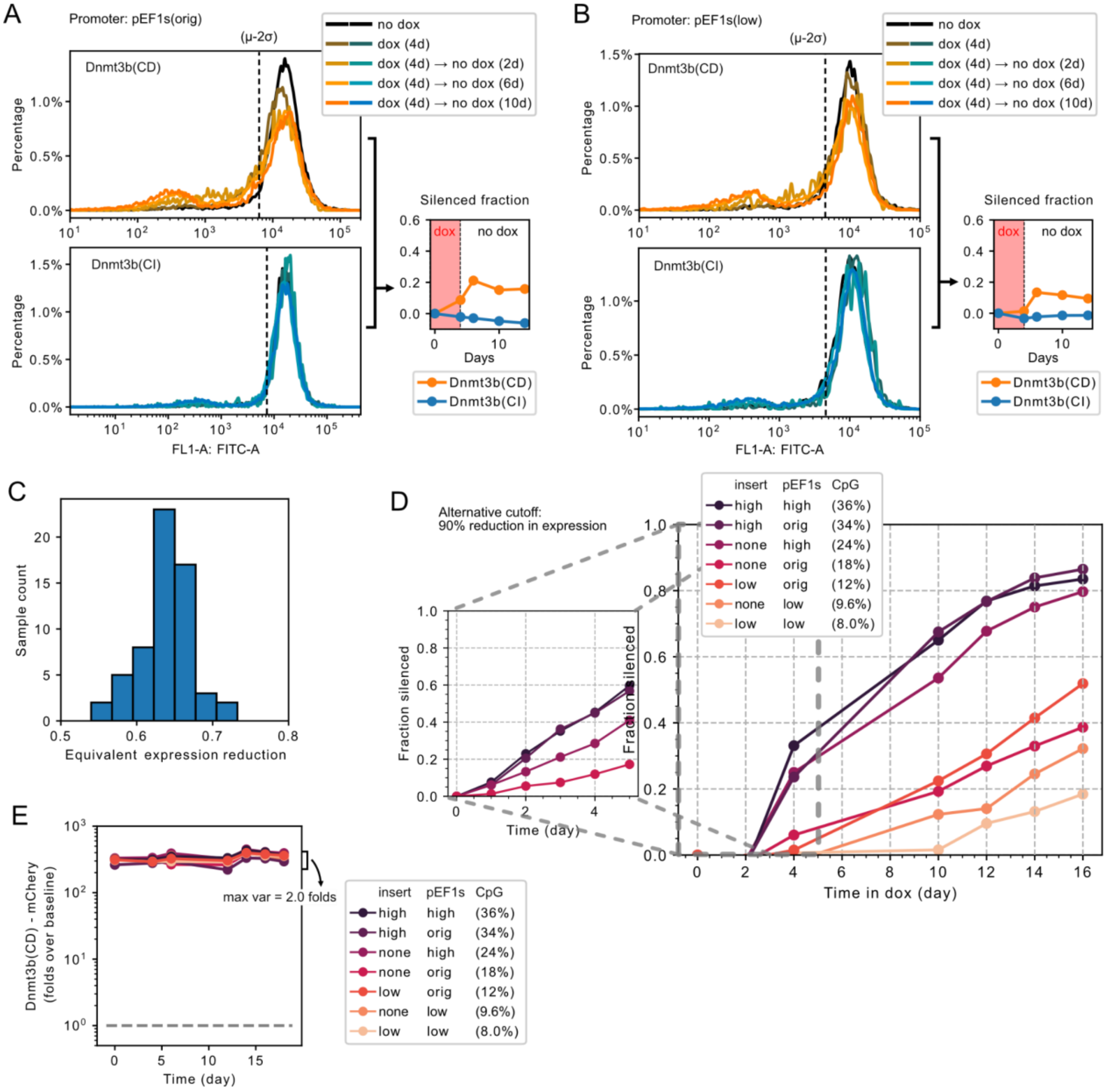
A
Promoter: pEF1s(orig)
(μ-2σ)
no dox
dox (4d)
dox (4d) → no dox (2d)
dox (4d) → no dox (6d)
dox (4d) → no dox (10d)
Dnmt3b(CD)
Dnmt3b(CI)
Percentage
FL1-A: FITC-A
Silenced fraction
dox
no dox
Days
Dnmt3b(CD)
Dnmt3b(CI)
B
Promoter: pEF1s(low)
(μ-2σ)
no dox
dox (4d)
dox (4d) → no dox (2d)
dox (4d) → no dox (6d)
dox (4d) → no dox (10d)
Dnmt3b(CD)
Dnmt3b(CI)
Percentage
FL1-A: FITC-A
Silenced fraction
dox
no dox
Days
Dnmt3b(CD)
Dnmt3b(CI)
C
Sample count
Equivalent expression reduction
D
Alternative cutoff:
90% reduction in expression
insert pEF1s CpG
high high (36%)
high orig (34%)
none high (24%)
none orig (18%)
low orig (12%)
none low (9.6%)
low low (8.0%)
Fraction silenced
Time (day)
Time in dox (day)
E
Dnmt3b(CD) - mChery
(folds over baseline)
max var = 2.0 folds
Time (day)
insert pEF1s CpG
high high (36%)
high orig (34%)
none high (24%)
none orig (18%)
low orig (12%)
none low (9.6%)
low low (8.0%)

Figure S2. Promoters' silencing rate correlates with their CpG content.

- *A-B. Promoter silences with all-or-none kinetics when DNMT3bCD is recruited to the locus, and this silencing is dependent on DNMT3b's catalytic activity. Same as Figure 2A except for promoter pEF1s(orig) (A) and pEF1s(low) (B). Lower silencing fractions were observed in general with these two promoters, but silenced populations remain stable after two days of dox release. DNMT3bCI does not silence either promoter.*
- *C. Comparison between the cutoff criteria we use (2σ from control expression, as in Figure 2 and Supplementary Figure S2A-B) to more traditional "expression reduction" criteria. Our criteria is equivalent to expression reduction from 54% to 71%, depending on the cell lines and time points, forming a single peaked distribution.*
- *D. Same time course as Figure 2B, with cutoff as 90% reduction in expression to determine silenced fraction. Though this method is less sensitive, similar correlation between promoter CpG densities and silencing rates are observed.*
- *E. DNMT3bCD expression level maintained stable throughout the time course (Figure 2B). The mCherry co-expressed with DNMT3bCD maintained around 300 folds over the baseline throughout the time course, with maximal variation of 2 folds across different cell lines and time points.*

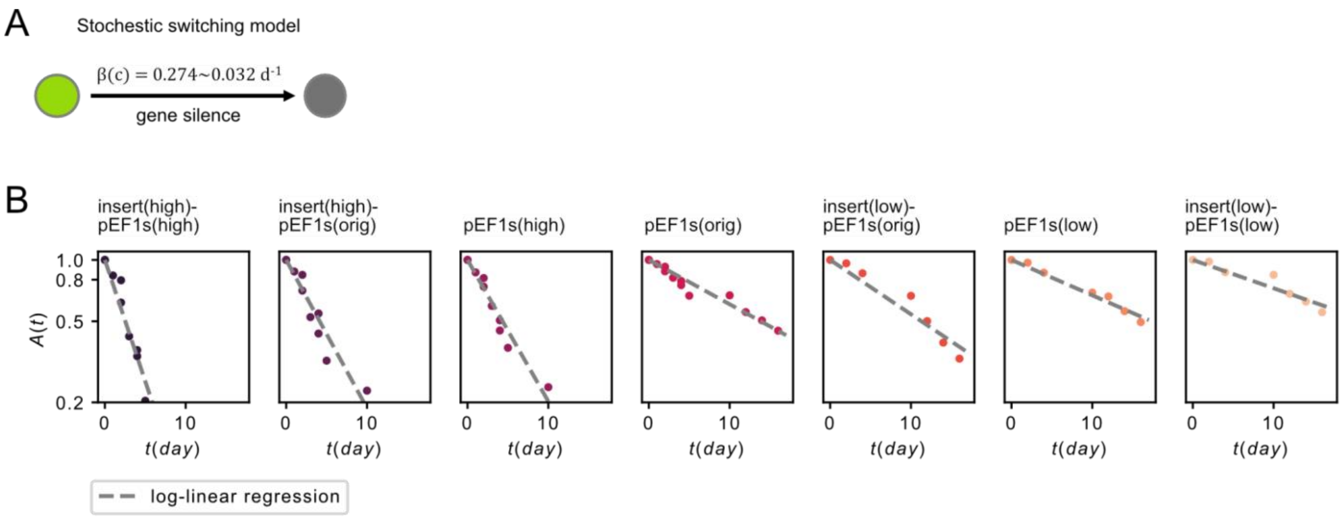

Figure S3: Promoters silencing dynamics follows a stochastic switching model.

A. *Stochastic switching model of the silencing dynamics (see also Eq. 1).*

B. *Time course from Figure 2B replotted as* $A(t)$ *(1 - silenced fraction) against time* t*. The* $A(t)$ *(y-axis) is plotted in log scale as the model predicts a linear-log relationship. Regression is performed with the least-square method.*

REFERENCES

(1) Weirauch, M. T.; Yang, A.; Albu, M.; Cote, A. G.; Montenegro-Montero, A.; Drewe, P.; Najafabadi, H. S.; Lambert, S. A.; Mann, I.; Cook, K.; Zheng, H.; Goity, A.; van Bakel, H.; Lozano, J.-C.; Galli, M.; Lewsey, M. G.; Huang, E.; Mukherjee, T.; Chen, X.; Reece-Hoyes, J. S.; Govindarajan, S.; Shaulsky, G.; Walhout, A. J. M.; Bouget, F.-Y.; Ratsch, G.; Larrondo, L. F.; Ecker, J. R.; Hughes, T. R. Determination and Inference of Eukaryotic Transcription Factor Sequence Specificity. *Cell* **2014**, *158* (6), 1431–1443.

(2) Kondratova, A.; Watanabe, T.; Marotta, M.; Cannon, M.; Segall, A. M.; Serre, D.; Tanaka, H. Replication Fork Integrity and Intra-S Phase Checkpoint Suppress Gene Amplification. *Nucleic Acids Res.* **2015**, *43* (5), 2678–2690.

(3) Nagaraj, N.; Wisniewski, J. R.; Geiger, T.; Cox, J.; Kircher, M.; Kelso, J.; Pääbo, S.; Mann, M. Deep Proteome and Transcriptome Mapping of a Human Cancer Cell Line. *Mol. Syst. Biol.* **2011**, *7*, 548.
